# Advances in Nano-Functional Materials in Targeted Thrombolytic Drug Delivery

**DOI:** 10.3390/molecules29102325

**Published:** 2024-05-15

**Authors:** Tengfei Ren, Yuexi Mi, Jingjing Wei, Xiangyuan Han, Xingxiu Zhang, Qian Zhu, Tong Yue, Wenhao Gao, Xudong Niu, Cuiyan Han, Bing Wei

**Affiliations:** 1 School of Basic Medical Sciences, Qiqihar Medical University, Qiqihar 161006, China; 202152745@stu.qmu.edu.cn (T.R.);; 2 School of Pharmacy, Qiqihar Medical University, Qiqihar 161006, China; 3 School of Materials Science and Engineering, Heilongjiang University of Science and Technology, Harbin 150022, China

**Keywords:** thrombosis, thrombolytic drugs, organic nanomaterials, inorganic nanomaterials, biomimetic nanomaterials, drug delivery system

## Abstract

Thrombotic disease has been listed as the third most fatal vascular disease in the world. After decades of development, clinical thrombolytic drugs still cannot avoid the occurrence of adverse reactions such as bleeding. A number of studies have shown that the application of various nano-functional materials in thrombus-targeted drug delivery, combined with external stimuli, such as magnetic, near-infrared light, ultrasound, etc., enrich the drugs in the thrombus site and use the properties of nano-functional materials for collaborative thrombolysis, which can effectively reduce adverse reactions such as bleeding and improve thrombolysis efficiency. In this paper, the research progress of organic nanomaterials, inorganic nanomaterials, and biomimetic nanomaterials for drug delivery is briefly reviewed.

## 1. Introduction

**Figure 1:**
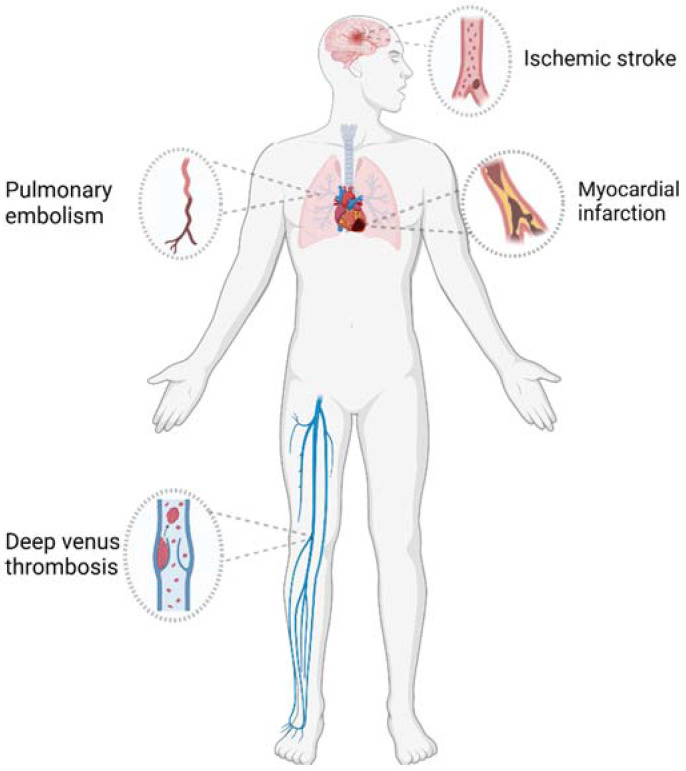
Schematic diagram of thromboembolic disease. Created with BioRender.com (accessed on 3 March 2024).

Thrombosis refers to the formation of a blood clot when visible components of the blood coagulate within the vascular system, thereby slowing or stopping blood flow, impacting the nutritional supply and gas exchange to tissues, leading to ischemic necrosis of the tissue and posing a life-threatening hazard. Based on the location of thrombus formation within the vascular system, thrombi can be categorized into arterial thrombi, venous thrombi, and microcirculatory thrombi. Arterial thrombi frequently result in ischemic heart disease and ischemic stroke (such as coronary heart disease and cerebral apoplexy) [1]. Venous thromboembolism (VTE) is divided into deep venous thromboembolism and pulmonary embolism (Figure 1). The annual incidence of VTE is 1–2 per 1000 people, making it the third most common vascular disease in the world [2]. As a country with a large population, China now has about 330 million patients with cardiovascular diseases, including 13 million stroke patients and 11.39 million coronary heart disease patients. In China, there are 48 patients with VTE per 100,000 people, and about 1.04 million patients with VTE in the United States in 2019, with a high incidence and mortality [3,4]. In 2020, the new coronavirus COVID-19 led to a global pandemic. Hundreds of millions of people around the world suffered from COVID-19, and this group of people had a higher incidence of VTE than those without it [5,6]. The induction of thrombotic disorders is multifactorial. Factors such as hormonal disturbances due to pregnancy, smoking and poor living habits like staying up late, comorbidities like cancer and autoimmune diseases, and aging can all lead to thrombosis [7,8].

In the treatment of acute thrombotic events, immediate administration of thrombolytic drugs is the best way to improve the survival rate in patients, but its short half-life, the need for multiple perfusion and easy-to-induce systemic hemorrhage are still problems to be solved in clinical treatment. Currently, there are the options of targeted open surgery and interventional therapy, but it is easy to damage the vascular intima and cause thrombosis again [9]. Therefore, people urgently need a safe, efficient, and economic way to treat thrombotic diseases. With the rise of nanotechnology, controlling the targeted delivery of thrombolytic drugs with nanocarriers is becoming a new force in the field of thrombus therapy, which can improve the efficiency and safety of thrombus therapy.

In this paper, the research progress of nano-functional materials in the field of thrombus diagnosis and treatment is reviewed. Chinese and English research articles in the field of thrombus diagnosis and treatment from the last 10 years are retrieved. The retrieval databases include “PubMed, ScienceDirect, Web of Science, Google Scholar”, and the retrieval keywords include “nanotechnology”, “thrombus diagnosis and treatment”, “magnetic guided nano delivery”, “biomimetic nano system”, “liposome” “nano silicon”, “polymer delivery”, “thrombosis”, “drug delivery system”, “targeted thrombolysis”, “thrombolytic drugs”, and other keywords. This paper will focus on the application of inorganic nanomaterials, organic nanomaterials, and biomimetic nanomaterials in this field, analyze their advantages and disadvantages according to their effects in vivo and in vitro experiments, and put forward prospects for their development.

### 1.1. Pathophysiologic Basis for Causing Venous Thrombosis

Venous thrombosis mainly comprises deep venous thromboembolism and pulmonary embolism (PE), and has become the third largest vascular disease in the world (after myocardial infarction and stroke). Under normal physiological conditions, there is a mutual restriction between anticoagulant components and coagulation factors in the body, so coagulation reaction will not occur. When the relationship is disturbed or destroyed, the pathological phenomenon of thrombosis in blood vessels will occur. In 1856, the famous doctor Rudolf Virchow first proposed the triad model of thrombosis mechanism, which attributed the development of clinical thrombosis to the combination of vascular endothelial cell injury, blood flow changes, and abnormal blood composition [10,11]. ① Vascular endothelial cell injury. The injury of vascular endothelial cells leads to abnormal expression of various anticoagulants, regulatory proteins and adhesion molecules, and the expression of tissue factors on activated endothelial cells, which starts the internal and external pathways of coagulation cascade reaction to promote blood coagulation and lead to thrombosis [12,13]. ② Changes in blood flow status. Under normal physiological conditions, the visible components in the blood flow around the central axis of the blood flow, while the plasma forms an edge flow in the outermost layer. When the blood flow rate slows down, the platelet enters the edge flow, which increases the chance of contact with the intima of the blood vessel and then adheres to the intima. At the same time, the existence of a vortex in the blood vessel will also accelerate the separation of platelets, deposit on the inner wall of the blood vessel after adhesion, and promote the formation of thrombosis. The decrease in blood flow and stasis lead to the accumulation of thrombin in the lesion, which overcomes the local anticoagulant pathway and induces thrombosis [14,15]. ③ Abnormal blood composition. The components of platelets and coagulation factors in the blood increase, the anticoagulant function decreases, and the abnormal release of fibrinolytic enzyme causes the blood to enter a hypercoagulable state of thrombosis, which leads to coagulation and thrombosis [16,17]. With further exploration of the mechanism of thrombosis, the damage to the structure and function of the largest number of red blood cells in the human body also directly or indirectly mediates the process of thrombosis. Red blood cells are unique deformable cells. When they pass through microvessels, their shape changes from double-concave disc shape to bullet shape. When their deformation ability becomes weak, they are easily blocked to form microthrombosis. Red blood cells produce a large number of intracellular reactive oxygen species (ROS) and accumulate extracellular reactive oxygen species through NADPH oxidase activation and hemoglobin autoxidation. The structure of red blood cell membrane changes in this oxidative environment, resulting in damage or fragmentation of red blood cell structure and changes in function. Abnormal red blood cells combine with endothelial cells, platelet and coagulation factor activation, phosphatidylserine exposure, and microbubble release to promote thrombosis. In addition, abnormal red blood cells adhere to the vascular wall to induce the production of thrombin in the thrombus and accelerate the formation of thrombi [18,19].

### 1.2. Clinical Treatment of Thrombotic Diseases

For cardiovascular disease caused by acute thrombosis, drug therapy, interventional therapy, and surgical treatment are usually used in clinics at this stage. Taking the refractory deep venous thromboembolism of the lower extremity (LEDVT) as an example, the treatment methods for the acute phase mainly include anticoagulation, intravenous active drugs, drug thrombolysis, inferior vena cava filter implantation (IVCFI), mechanical thrombectomy, etc. [20]. When deep venous thrombosis (DVT) is found or suspected, anticoagulants should be given priority to inhibit the expansion and spread of the thrombus plate, and then corresponding thrombectomy or thrombolysis methods should be used to eradicate the thrombus [21]. For patients who need urgent thrombectomy, open surgery or minimally invasive interventional surgery can be used clinically. During open surgery through control of the target vein and incision, the thrombus will be squeezed out or removed by using instruments, but this treatment will damage the intima of blood vessels, which is very easily causes the reformation of the thrombus. Compared with open surgery, interventional therapy is safer and more effective. It is widely used in clinics because of its high safety and good curative effect. It can be directly aspirated or broken and dissolved by using a large lumen catheter, or it can also be a highly effective thrombolysis by injecting thrombolytic agent through a catheter after catheterization. The more direct treatment of thrombotic disease is the use of injection or oral thrombolytic drugs to promote the dissolution of fibrin. However, there are many limitations, such as severe trauma, thrombolytic drug allergy, other cardiovascular diseases that may be life-threatening, the short half-life of thrombolytic drugs, and an inability to accumulate a large number of thrombolytic drugs in the thrombus for efficient thrombolysis, which prevent this method from becoming the optimal thrombolytic treatment [22].

Currently, thrombolytic drugs used in clinical practice are divided into three generations. The first generation of thrombolytic drugs is represented by urokinase (UK) and streptokinase. Streptokinase is a single-peptide multi-chain that indirectly plays the role of fibrinolysis by activating plasminogen in the blood circulation. This drug has been approved by the FDA for acute progressive myocardial infarction, pulmonary embolism, deep vein thrombosis, and arterial thrombosis, but its clinical application is limited because it is an exogenous protein prone to immune reaction. Urokinase, also known as urokinase-type plasminogen activator, is a serine protease produced by renal parenchymal cells, which can convert plasminogen into plasmin. Fibrinolytic enzyme can directly degrade fibrin in thrombi and dissolve thrombi. Because urokinase has no fibrin specificity and a short half-life, it needs long-term continuous infusion. It is usually combined with heparin anticoagulation in clinics. The second generation of thrombolytic drugs include prourokinase, tissue plasminogen activator (t-PA), and alteplase. Compared with first-generation thrombolytic drugs, the ability and half-life of targeting fibrin in thrombi are increased. Alteplase is widely used in the intervention of acute ischemic stroke and acute pulmonary embolism in developing countries such as India [23,24]. Third-generation thrombolytic drugs include reteplase and teneplase. Some of these drugs have variants of t-PA. Through structural changes or modifications, they have a longer half-life, higher safety, and stronger fibrin specificity. Compared with the second generation, they have a lower risk of systemic hemorrhage. In the treatment of acute ischemic stroke, compared with alteplase, the use of teneplase can reduce the incidence of cerebral hemorrhage [25,26,27]. The pharmaceutical formulations that can be directly used for thrombolysis are still under research and development. A study conducted by Planer et al. showed that catheter-guided thrombolysis (CDT) has significant advantages over systemic thrombolysis and anticoagulation therapy. CDT therapy is essentially local thrombolysis and does not affect other sites [28]. However, catheter-directed thrombolysis requires the patient to enter the interventional radiology laboratory quickly for catheter insertion and needs to be implemented by experienced doctors. When early recanalization is required, low-dose thrombolysis is more attractive to patients [29,30]. Because nanoscale phenomena play an important role in signal transduction at the cellular and subcellular levels, nanotechnology provides tools and technologies for the transfer of fine organic macromolecules and peptides, preventing them from being cleared by the immune system, allowing for them to pass through the barrier that prevents macromolecules from passing through, accurately targeting and acting on the target location of the disease [31,32]. Drug delivery with nanocarriers is similar to CDT therapy. With the rise and development of nanotechnology, nanothrombolytic delivery is expected to pass clinical trials and enter clinical application. When catheter thrombolysis is limited, nanodelivery may become one of the best treatment methods.

## 2. Application of Nano-Functional Materials in Targeted Drug Delivery

### 2.1. Development of Inorganic Nanomaterials in Drug Delivery

**Figure 2:**
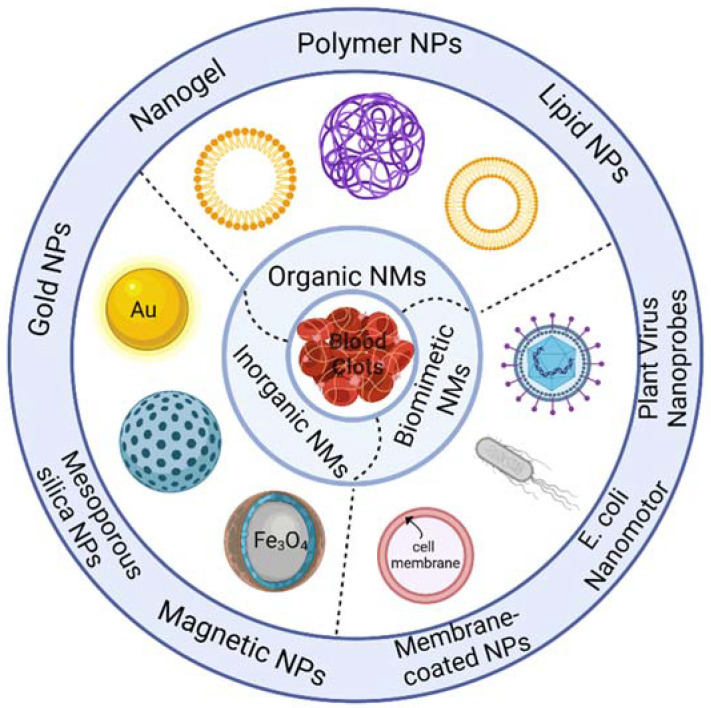
Schematic of a nanocarrier for delivery of thrombolytic drugs for thrombolysis. Created with BioRender.com (accessed on 3 March 2024).

Currently, numerous researchers internationally have developed a variety of nanocarriers for dissolving thrombi, and by utilizing the inherent properties of nanocarriers in conjunction with external stimuli, they can synergistically dissolve thrombi. In this paper, we summarize the current nano-functional materials used for delivering thrombolytic drugs in Figure 2.

#### 2.1.1. Targeted Drug Delivery of Magnetic Nanomaterials

**Figure 3:**
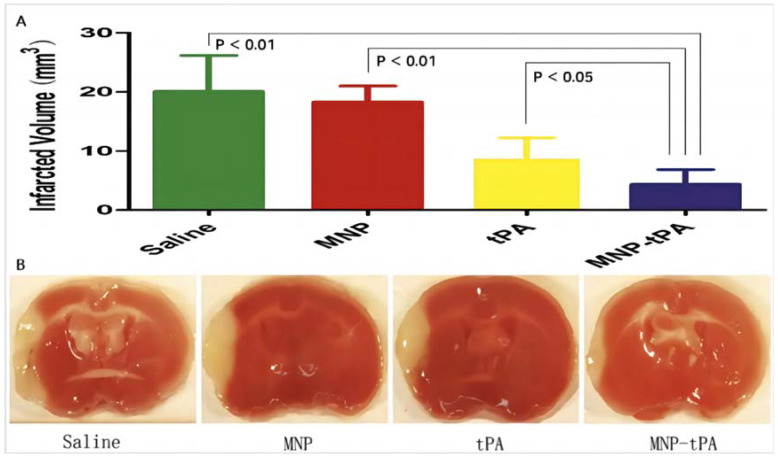
(**A**) After the injection of four different reagents into the thrombus model, the infarct area demonstrated that MNP-rtPA has a significant thrombolytic effect. (**B**) Representative images of 2,3,4 triphenyltetrazolium chloride (TTC) staining. Reproduced with permission [36]. Copyright 2019, Elsevier.

Magnetic nanoparticles (MNPs) can undergo directional motion under the action of an external magnetic field. When the external magnetic field is removed, they can be uniformly dispersed again in solution. In addition, magnetic nanoparticles have characteristics such as large specific surface area, stable chemical properties, and good biocompatibility, making them widely used in the biomedical field. By functionalizing the surface of magnetic nanoparticles, they can be applied to the early diagnosis and treatment of cardiovascular diseases and cancer [33]. However, there are few magnetic nanoparticles combined with magnetic resonance imaging (MRI) for early diagnosis of cerebral infarction. Hu et al. developed an enhanced fluorescent protein EGFP-EGF1 that targets the formation of cerebral thrombosis caused by overexpression of tissue factor (TF) and combined it with superparamagnetic iron oxide (Fe_3_O_4_) for early diagnosis of cerebral thrombosis. Through fluorescence imaging and pharmacokinetic analysis, the feasibility of the nanoparticles in the early diagnosis of cerebral thrombosis was further demonstrated [34]. Superparamagnetic nanomaterials have defects such as easy aggregation and weak drug loading ability. Usually, polymer or other material coatings are applied to the surface of magnetic nanoparticles to suppress their aggregation, and improve stability and drug loading [35]. Huang et al. coated the surface of MNPs with polyacrylic acid (PAA) to prevent MNP aggregation and used covalent binding to recombine tissue plasminogen activator (rtPA is the only thrombolytic agent approved by the FDA for the treatment of ischemic stroke) to bind to PAA-MNP. In vitro research experiments have shown that the mechanical drag force generated by the external magnetic field on the nanoparticles enhances the thrombolytic effect. This indicates that the application of PBS, MNP, rtPA, and MNP-rtPA reagents to middle cerebral artery thrombosis in mice resulted in an increase in infarct size of 20.09 ± 6.07, 18.28 ± 2.698, 65 ± 3.63, and 4.40 ± 2.46 mm^3^ (Figure 3A,B), indicating that MNP-rtPA significantly reduced infarct size compared to other groups [36]. Compared with single-target drug delivery systems, nanoparticles with dual or multiple targets can deliver drugs more accurately to the lesion location [37]. Chen et al. further developed a drug delivery system for dual targeted delivery of rtPA. The rtPA and the fiber protein affinity peptide (GPRPPGGSKGC) were co-grafted onto poly(lactic-co-glycolic acid) (PLGA), and then this co-assembly was combined with MNPs to prepare pPMNP-rtPA, thereby implementing a dual targeting strategy that combines magnetic guidance with the target peptide. pPMNPs exhibit high affinity for thrombi in multimodal fluorescence imaging and MRI of intravascular thrombi. In vitro dynamic experiments showed that the combination of magnetic guidance and targeted peptides achieved the shortest reperfusion time, which increased lysis efficiency by 2.3 times compared to the pPMNP group. In the left iliac artery thrombosis model of rats, the blood flow downstream of the blood clot was restored 35 min after the injection of pPMNP-rtPA reagent at a dose of 0.3 U/kg, indicating the feasibility of a dual targeted drug delivery therapy strategy for future clinical thrombus treatment [38].

The dual-directional magnetic nanoparticles improved the transport ability of rtPA targeting the thrombus, but the responsive release ability after reaching the location of the thrombus lesion was not correspondingly improved. To address this need, Wang et al.’s research team developed a nanoparticle shell microbubble composed of an air core, magnetic nanoparticles, and mesoporous silica nanoparticles as shells. By carrying drugs through mesoporous silica, magnetic nanoparticle shells assist microbubbles in targeting blood clots under magnetic field guidance. Utilizing ultrasonic vibrations to excite microbubbles, the oscillation of the microbubbles releases encapsulated nanoparticles, imparting kinetic energy to them. These nanoparticles can penetrate thrombi as large as 1 micrometer, enhancing the efficiency of thrombolysis. When the nanoparticle-coated microbubbles are used to treat thrombi in vitro, they exhibit an astonishing dissolution rate of approximately 93%. In a mouse femoral vein thrombosis model, compared to conventional injection of tPA [tPA (0.03 mg/kg)], the injection of nanoparticle microbubble tPA [tPA (0.03 mg/kg)] resulted in a reduction of nearly 67.5% in residual femoral vein thrombosis. This treatment strategy may provide more possibilities for the treatment of acute thrombosis [39].

Thrombosis leads to ischemic damage to tissues, and although thromboembolism is successfully eliminated, ischemic damage to downstream tissues continues [40,41]. Therefore, there is an urgent need for a thrombus treatment strategy that can successfully unblock blood vessels and save ischemic tissue damage. Xu et al. constructed a multifunctional integrated thrombus diagnosis and treatment platform based on magnetic response. The platform combines bioactive ion therapy (with properties such as repairing damaged tissues and stimulating angiogenesis) with magnetic response nanomedicine delivery strategies. Polyamine (PDA) is used as a linker to connect magnetic nanoparticles with third-generation thrombolytic drugs. An external magnetic field is applied to quickly enrich the thrombus site and release thrombolytic drugs to achieve efficient thrombolysis. When ischemia–reperfusion injury is restored, Sr ions (bioactive ions) cooperate with magnetic nanoparticles to clear reactive nitrogen and oxygen (RONS), treat ischemia–reperfusion injury, and repair damaged tissues. The PDA used has good photoacoustic signals and exhibits excellent detection performance in the renal artery thrombosis model, which is used for detecting thrombosis and monitoring the thrombolysis effect. Compared with a single thrombolytic drug, the integrated drug-loaded nanoparticles have a 3.04-fold increase in drug half-life and a 35.18% reduction in thrombus residue rate in the occlusive thrombus model, significantly prolonging the drug half-life and accelerating thrombus dissolution. By analyzing the levels of blood urea nitrogen (BUN) and creatinine (CRE) in renal tissue of mice with ischemia–reperfusion injury, it was verified that this integrated nanoparticle can significantly reduce BUN and CRE levels and restore mouse kidney function. This provides an excellent treatment strategy for tissue damage after thrombotic diseases [42].

Light has the characteristics of noninvasiveness, good spatiotemporal controllability, and ease of application. The application of light in the diagnosis and treatment of diseases has great potential. Photodynamic therapy (PDT) and photothermal therapy (PTT) have been developed for the treatment of diseases such as thrombosis, tumors, and cancer [43,44,45,46,47]. Most thrombolytic drugs used for PTT and PDT therapy can not accurately accumulate at the thrombus site, so Liu et al. designed a multimodal imaging guided combination mechanical phototherapy thrombolysis method with multiple targets and integrated diagnosis and treatment, which self-assembles human neutrophil lysate (Nu), fucoidan (Fu), iron oxide (IO), and methylene blue (MB) through plasma (an ionized gas used for sterilization, cancer treatment, and regenerative medicine), forming micelle nanoclusters. Methylene blue is used as a PDT reagent and fluorescent tracer, while Nu and Fu are used to target thrombus clots. In the mouse model of thrombosis, the residual thrombus formation was reduced by 80% under NIR irradiation and magnetic guidance, and there was no risk of re-embolism. There is a clear prospect for treating life-threatening cardiovascular diseases [48]. Magnetic nanoparticles, visualized by MRI, can be integrated with clinical imaging modalities for precise localization of thrombus locations. The combination of ultrasound and microbubbles enhances the permeability of the nanoparticles in blood clots, offering a solution for acute thrombotic diseases. Currently, this ultrasound–microbubble-based thrombolysis strategy has entered clinical trials and demonstrated promising efficacy. This approach is expected to be clinically implemented in the future. The multifunctional integrated diagnostic and therapeutic platform based on magnetic design not only exhibits excellent thrombolytic effects but also integrates imaging tracking and monitoring of thrombolytic efficacy, holding significant potential for clinical translation.

#### 2.1.2. Mesoporous Silica Nanoparticle Delivery

Mesoporous silica is widely used in chemical industry, energy, environmental engineering, biomedicine, and many other fields because of its stable structure, easily controlled pore size, large specific surface area, easy surface modification, and other advantages [49,50]. In 1992, Beck et al. were the first to synthesize mesoporous silica materials with an ordered two-dimensional hexagonal pore structure using the sol–gel method [51]. In 2001, Vallet-Reg et al. were the first to load ibuprofen with MCM-41 mesoporous silica nanoparticles (MSNs) as a drug carrier, showing a high drug loading rate and successfully released ibuprofen [52]. Since then, MSNs as a carrier for drug delivery have set off an upsurge in biomedical research. As drug carriers, MSNs have a high drug loading rate and excellent characteristics of preventing drug crystallization due to their unique pore structure. At the same time, MSNs have two sides, and are as prone to drug leakage as other nanocarriers. Xu et al. constructed a drug delivery system using fatty acids to plug a gold mesoporous silica core–shell structure. Fatty acids composed of lauric acid and stearic acid are used as phase-change materials to block the pores, which can degrade at a specific temperature, and the degraded products can reduce the risk of atherosclerosis. The Au mesoporous silica core–shell structure can induce a luminescence thermal effect under near-infrared light irradiation. On the one hand, the heat generated is used to make fatty acids undergo phase change reaction and release thrombolytic drugs encapsulated in mesoporous silica. On the other hand, local high temperature can promote the dissolution of the thrombus. The study proved its thrombolytic effect through in vivo and in vitro experiments. In vitro, it was successfully proved that the structure had the best dissolution of blood clots in dynamic and static liquid, and the thrombolysis speed was nearly 30 min faster than that of the free group. In the established rabbit femoral vein thrombosis model, the treatment group with this structure showed a better dissolution effect than other treatment groups, and the thrombus was almost completely dissolved the next day [53]. Due to the lack of thrombus targeting, the utilization rate of nanoparticles for the delivery of thrombolytic drugs is low. To solve these problems, Chen et al. used the high drug loading rate of silicon dioxide and the characteristics of Fe_3_O_4_ being guided by magnets or magnetic fields to prepare nanocarriers composed of a superparamagnetic iron oxide core (Fe_3_O_4_) and silicon dioxide shell. In this study, the magnet was placed under the clot for magnetic targeting. The magnetically guided MSNs were 66% of non-magnetic operation and 60% of free TPA operation. The combination of the two nano materials enhances the permeability to blood clots and effectively shortens the thrombolytic time [54]

In addition to thrombus targeting through physical methods, the ability of nanoparticles to target thrombi can also be improved by modifying molecules such as peptides on the surface of nanomaterials, and peptides can target specific sites of thrombi through antigen antibody binding (such as how RGD peptide can specifically target the glycoprotein GPIIb/IIIa receptor of an activated platelet membrane) [55]. Huang et al. studied and prepared mesoporous silica nanoparticles with dual targeting ability. The research team used magnetic mesoporous silica as the core of nanoparticles, combined polyglutamic acid dendrimers with nanoparticles, and finally grafted arginine glycine aspartate peptide (RGD) onto nanoparticles, enhancing its ability to actively target thrombi. Using the electrostatic adsorption between the positively charged and negatively charged peptide dendrimers of nattokinase (NK), the drug loading of nanoparticles on NK was increased, so as to improve the thrombolytic efficiency of NK. In an in vitro thrombolysis experiment, the weight of the thrombus in the RGD peptide-containing group and non-RGD peptide passive targeting group decreased from 100% to 70% and 55.3%, respectively, after 5.5 h. In the rat carotid artery model, compared with the passive targeting group, the nanoparticles with this structure accumulated more at the site of thrombus lesions, and the thrombus contrast became porous before treatment, which showed that the nanoparticles modified with targeted peptides on the surface improved thrombolysis efficiency [56].

**Figure 4:**
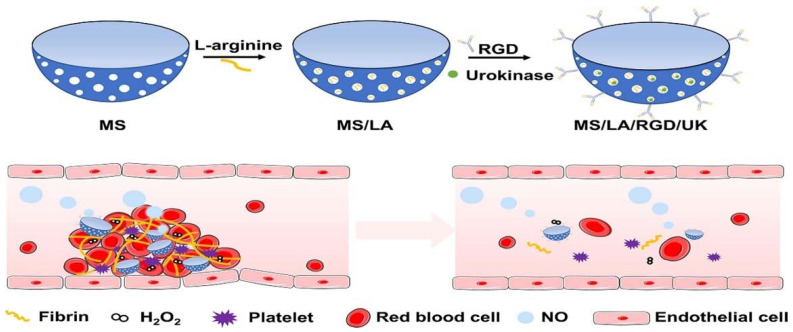
Schematic of the synthesis of asymmetric-structured bowl-shaped mesoporous silica nanoparticles and their thrombolytic process. Reproduced with permission [60]. Copyright 2021, Elsevier.

Because the blood flow speed is too fast, thrombolytic drugs cannot stay in the thrombus for a long time, which makes the drug utilization rate low. With the progress of micro/nanotechnology, micro/nanomotors, which can convert external energy into internal power, have attracted the attention of biomedical researchers. Loading micro/nanomotors with active motion capability on nanocarriers can overcome the limitations of traditional passive drug delivery nanoparticles and realize the precise drug delivery of a nanodelivery system within a specific space and time [57]. Tao et al. developed and designed bowl-shaped mesoporous silica nanoparticles with asymmetric structure. Using silica as the substrate, L-arginine (LA) and UK were loaded into the pores at the same time. The RGD peptide was modified on the surface of the bowl-shaped mesoporous silica nanoparticles to target the thrombus lesions. There are excessive reactive oxygen species (ROS) produced by damaged endothelial cells and activated platelets at the thrombus site. ROS can aggravate the damage of endothelial cells and activation of platelets. Activated platelets can produce H_2_O_2_, which can produce NO through corresponding reaction (NO can repair damaged endothelial cells, and the generated NO can also be used as the power source of the nanomotor [58,59]). On the one hand, LA can act as a gated channel to control the release of UK; on the other hand, it can react with excessive reactive oxygen species (ROS) at the thrombus site, reducing the inflammatory response of endothelial cells (Figure 4). The study reported that this kind of nanomotor moves at 3.52 microns per second in the thrombus microenvironment. In vitro and in vivo experiments showed an excellent thrombolytic effect. In vitro experiments revealed that the nanomotor group achieved a significant increase in fibrinolysis, with a rate of up to 74%. This was followed by a further nearly 20% improvement in the area of fibrinolysis compared to the non-nanomotor group. After seven days of thrombolytic therapy, the relative thrombus volumes in rats treated with UK, non-nanomotor, and nanomotor groups were reduced to 0.62, 0.35, and 0.20, respectively. Notably, the nanomotor group demonstrated the best thrombolytic efficacy. It was detected by fluorescence imaging that the nanomotor group was enriched at the thrombus site, indicating that the nanocarrier with active motion can enhance the retention rate at the thrombus site [60]. The design of this structure not only enhances the efficiency of thrombolysis but also further repairs damaged endothelial cells. However, there is an issue: although the mesoporous silica nanoparticles are endowed with motility to increase their high concentration at the thrombus site, the favorable thrombolytic effect relies on a prolonged treatment duration. This indicates that nanoparticles with this structure are not suitable for emergency treatment of thromboembolic diseases in the acute phase.

#### 2.1.3. Other Inorganic Nanomaterials for Thrombus Therapy

As we all know, bulk gold used in daily life has good “safety” and chemical inertia. Gold visible to the naked eye is gold, but gold at the nanoscale can show red, blue, green and brown. At the same time, due to its local surface plasmon resonance, it can efficiently absorb near-infrared light and realize light conversion to heat. Such characteristics mean that gold nanoparticles show great potential as photothermal therapeutic agents and imaging agents in biomedicine [61,62]. In 2015, due to the shortcomings of the Cy5.5 near-infrared fluorescence (NIRF) probe, which cannot be discharged after cerebral thrombosis imaging and has no transformation potential for humans, Kim et al. reported, for the first time, the generation and characterization of gold nanoprobes for computed tomography (CT)-based direct cerebral thrombosis imaging, so as to detect thrombotic diseases and monitor the thrombolytic effect of tPA, and quantify the thrombus load [63]. In 2016, Singh et al. reported, for the first time, that gold nanorods exposed to near-infrared radiation were used for photothermal thrombus ablation. Remarkably, when streptokinase and NIR-irradiated gold nanorods were exposed to arterial and venous blood vessels containing blood clots in vitro, it was found that such synergistic therapy increased the thrombus ablation rate to 40.9% ± 5.38% and 19.5% ± 3.14%, respectively; this study preliminarily verifies the feasibility of photothermal thrombolysis treatment [64]. Although near-infrared light-mediated photothermal thrombolytic therapy overcomes the uncontrollable bleeding risk caused by thrombolytic agents to some extent, the thrombus fragments (>10 μm) produced after photothermal therapy may cause secondary embolism of microvessels. Zhang et al. proposed a specific dual-mode thrombolytic therapy combining photothermal therapy and photodynamic therapy. RGD was modified on mesoporous carbon nanospheres (PMCS) containing porphyrin-like metal centers to form nanoparticles (RGD-PMCS) that can produce heat and release ROS for specific thrombolysis under near-infrared irradiation (Based on the study that excessive ROS can inhibit the recurrence of thrombus by destroying lecithin platelet factor 3 (PF3) through lipid peroxidation [65]), the detection of PF3 in cells with different concentrations of RGD-PMCS exposed to an 808 nm laser for a certain period of time found that the amount of PF3 decreased sharply with the increase in RGD-PMCS concentration. The targeting ability of RGD was evaluated by using carbon-based nanoparticles as a light (PA) contrast agent. The results showed that RGD-PMCS could accumulate rapidly at the site of thrombosis within 1 h after intravenous injection, which was about 5 h compared with nanospheres without RGD. Histopathological evaluation showed that there was almost no thrombosis in the blood vessels of the near RGD-PMCS+NIR treatment group. Magnetic resonance imaging (MRI) verified the recanalization effect. Compared with the normal blood vessels, the revascularization rate was 87.9%. The blood clot penetration of RGD-PMCS under near-infrared light irradiation was about 26.3 mm. The overall experiment shows that the designed dual-mode treatment mode can not only remove thrombi with hyperthermia and ROS and prevent re embolism of small blood vessels, but also greatly improve thrombolytic efficiency by enhancing permeability, with a good trend of clinical transformation [66]. If the thrombus is embolized in the brain, excessive ROS produced during thrombolysis will lead to neuron damage [67]. Black phosphorus nanosheets (BPNs) have a high specific surface agent and negative charge, and can be loaded with drugs, targeted molecules, photosensitizers, etc. Their wide near-infrared absorption makes them candidates for photothermal therapy (PTT) and photodynamic therapy (PDT) for cancer and other diseases [68]. The excellent photothermal effect of BPNs under NIR irradiation can increase the blood–brain barrier permeability of BP, which provides a new neuroprotective platform for the treatment of neurodegenerative diseases (ND) [69]. Wang et al. used electrostatic interaction to load urokinase on BPNs. When the BPNs-uPA reached the thrombus position, uPA was released. At this time, the remaining BPNs penetrated the blood–brain barrier under 808 nm laser irradiation, removing excess ROS to protect neurons. In vitro, the BPNs-uPA dissolved more than 50 mg of thrombus after 2 h. Evans blue (EB), which is used to evaluate the blood–brain barrier, was injected into the tail vein of rats with BPNs in vivo. Three hours later, mice receiving BPN injection and laser irradiation were severely stained with Evans blue, while Evans blue was not observed in the brain of mice without laser irradiation, which confirmed that the photothermal properties of BPNs enhanced the permeability of the BBB. Then, mice with middle cerebral artery obstruction received BPNs-uPA injection and laser irradiation, the infarct percentage decreased by 28% compared with that at the beginning of establishment, and the neurological score was improved accordingly. These experimental results all prove that BPNs-uPA have an excellent neuroprotective effect on ischemic stroke [70].

### 2.2. Targeted Drug Delivery of Organic Nanomaterials

#### 2.2.1. Liposome Drug Delivery

**Figure 5:**
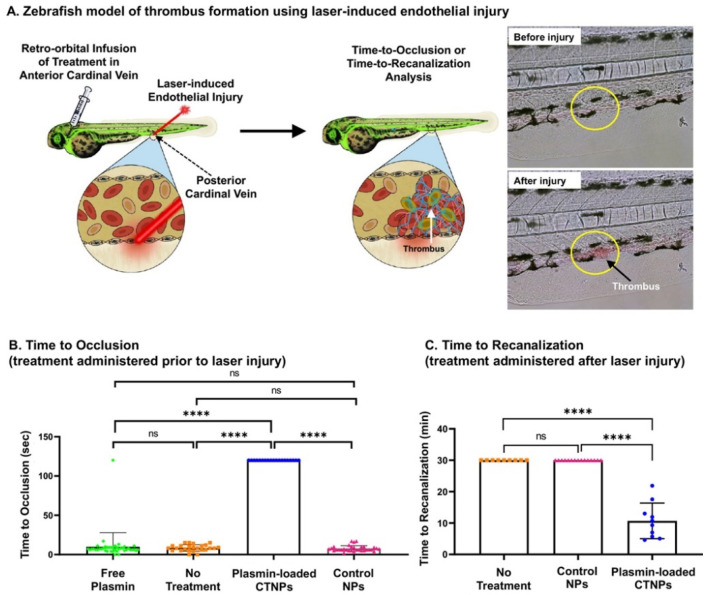
(**A**) Experimental setup for plasmin-loaded CTNP evaluation in the zebrafish venous thrombosis model, with representative brightfield images showing clot formation after laser injury; (**B**) time-to-occlusion (TTO) study showed that CTNPs loaded with plasmin could significantly prevent the formation of blood clots; (**C**) time-to-recanalization (TTR) study showed that CTNPs encapsulated with plasmin could achieve effective vascular recanalization within 20 min. Reproduced with permission, **** *p* ≤ 0.0001; ns not significant. [75]. Copyright 2022, Elsevier.

Liposomes are hollow spheres formed by lipid bilayer membranes. Lipids are one of the important components of organisms. Therefore, when they enter the human body in various forms, they do not cause strong immune response. Based on their good biocompatibility, low immunogenicity, and hollow structure, liposomes can be used as drug carriers to deliver corresponding drugs to treat various diseases [71]. In 1971, Ryman et al. used liposomes as drug carriers to deliver drugs in Britain [72]. Since then, biomedical researchers have begun to study liposome drug delivery. In the study by Zhang et al., cyclic RGD peptide (cRGD)-functionalized liposomes modified with polyethylene glycol exhibited superior thrombus targeting capabilities and thrombolytic effects. cRGD liposome effectively prolonged the time of urokinase in blood circulation. The thrombolytic effect of urokinase-coated cRGD liposomes was equivalent to that of four times the dose of free urokinase. The liposome containing urokinase cRGD provides a reference for the treatment of thrombosis in developed countries such as Europe and the United States [73]. For the treatment of thrombotic diseases, tPA is usually used to act on fibrinogen-related plasminogen, which is converted into plasmin to dissolve fibrin. However, this treatment method will not only act on the clot site, but also act on circulating plasminogen, causing adverse reactions such as bleeding. However, fibrinolytic agents acting directly on fibrin by oral or intravenous injection are easily neutralized by antiplasmin in vivo. Therefore, it is particularly important to construct a targeted drug delivery system that delivers plasmin directly acting on the fibrous units at the clot [74]. Sun et al. developed liposome-targeted nanoparticles (CTNPs) that can deliver plasmin, which is triggered by thrombin at the clot site, and the lipid membrane degrades and releases drugs. The heteromultivalent surface modification of nanoparticles can target the binding peptide of activated platelets (CGSSSGRGDSPA) and fibrin binding peptide (AC-Y (DGI) C (HPR) YGLCYIQGK-Am) for double-targeted thrombus clots. Thrombin-cleavable peptide (DVTPRC) was combined with nanoparticles to achieve thrombin-responsive degradation. The ability of CTNPs to target clots at blood flow-related shear force (25 dyn/cm^2^) was tested by microfluidic simulation. The confocal fluorescence image and Rhodamine B (RhB) fluorescence intensity analysis after 30 min showed that the double binding of platelet binding and fibrin binding made the CTNPs have higher specific targeting ability. In the zebrafish model of main vein thrombosis, the thrombolytic effect of CTNPs loaded with plasmin in vivo was analyzed by time-to-occlusion (TTO) and time-to-recanalization (TTR). The unprocessed nanoparticles rapidly occluded the blood vessels within 25 s, while the CTNPs loaded with plasmin did not cause vascular occlusion within 120 s. The TTO experiment showed that CTNPs loaded with plasmin could significantly inhibit the formation of thrombosis (Figure 5A,B). The control nanoparticles showed no signs of recanalization within 30 min, while the plasmin-loaded CTNPs achieved recanalization within 20 min. TTR experiments showed that the nanoparticles could effectively dissolve fibrin and re-dredge blood vessels (Figure 5C). This method provides an effective reference for the direct use of plasmin for fibrinolytic therapy [75]. However, the fibrinolytic enzyme in the nanoparticles can be released to a high degree only when thrombin-triggered instability occurs. The fibrinolytic enzyme released by the dispersion type can be easily neutralized by the circulating antiplasmin. Therefore, it is particularly important to design a kind of liposome nanoparticle that can release drugs under external control. Fan et al. designed and prepared an ultrasound-responsive nanoliposome capsule, which connected the ultrasound-stimulated release of ROS with the nanoliposome. When the nanoparticles reached the embolic position, the liposome containing the sonosensitizer produced ROS under the action of ultrasound, destroyed the sensitive bond in the liposome, and released urokinase from the cracked nanoliposome capsule and concentrated it on the clot position. In the rabbit pulmonary embolism model, most of the blood vessels were recanalized after 15 min with ultrasound-responsive nanoliposomes loaded with urokinase, which had twice the thrombolytic efficiency of free urokinase [76]. Sonosensitizers are stimulated by ultrasound to produce ROS. Although ROS destroy the structure of liposomes and release urokinase to achieve efficient thrombolysis, the excessive production of ROS will aggravate the injury of the endothelial system and increase the risk of thrombosis.

Laser is widely used in the biomedical field because of its high energy, high direction, high brightness, and other advantages. It can be used to separate tissues, ablate blood clots, trigger tumor cell apoptosis, for lithotripsy, and for other operations to treat related diseases by using lasers with different wavelengths and different powers. Among various laser types, the Nd:YAG laser is used in the treatment of oral diseases and thrombotic diseases because of its simple operation and high safety [77,78,79]. Ahmaditabar et al. designed a liposome drug delivery system (Lip/PSCs-tPA) with laser-controlled release of tPA for the treatment of arterial thromboembolic diseases. Deacetylated chitosan polysulfate (PSCs, with excellent biocompatibility and anticoagulant activity) was coated on nano liposomes containing tPA, and a Nd:YAG laser (532 nm) was used to achieve controlled release of tPA and targeted delivery of drugs to the thrombus. Laser beam irradiation of thrombi produces vacuoles, which makes the thrombus form a porous channel-like structure, similar to the tumor EPR effect. Nanoliposomes can penetrate into the thrombus to dissolve the thrombus better. By coating PSCs on the surface of nano liposomes, tPA can be released slowly and continuously, which can better prevent re-occlusion of blood vessels. In the dynamic thrombolysis experiment in vitro, due to the bubble generated by laser irradiation and the optomechanical force of the laser beam, nanoliposomes penetrated the thrombus, and the percentage of thrombus weight loss treated with Lip/PSCs tPA was about seven times that of free tPA. Through reverse-transcription–polymerase chain reaction (RT-PCR), the expression of tumor necrosis factor-α (TNF-α) and interleukin-10 (IL-10) genes in epithelial cells was quantitatively analyzed. The results showed that the expression of IL-10 in Lip/PSCs tPA group was significantly higher than that in the control group, and the expression of TNF in the Lip/PSCs tPA group was significantly higher than that in control group-α. The expression level of IL-10 was lower than that in the tPA group. The increase in IL-10 and decrease in TNF-α can improve myocardial injury and then treat coronary heart disease. In the rat femoral vein formation model, the percentage of thrombus area in the Lip/PSCs tPA group was 40% lower than that in the tPA treatment group, showing excellent thrombolytic ability [80]. The ultrasonic-responsive liposomes enhance the drug release rate and thrombus penetration effect. Non-contact laser stimulation induces a porous structure in the thrombus, thereby prolonging the retention time of nanoparticles at the thrombus site, allowing for the drug to act on the thrombus for an extended period, and enhancing the thrombolytic effect. However, it is necessary to consider the applicability of this approach in thromboembolic diseases, where the porous structure of the thrombus can facilitate the transformation of larger thrombi into smaller blood clots that can block smaller blood vessels, leading to hemorrhage in such areas. Therefore, this nanostructure model may not be suitable for clinical diseases involving large vascular embolism, such as lower-limb femoral vein embolism. We speculate that the low immunogenicity of multi-modified liposomal nanoparticles may be attributed to their early entry into clinical thrombolysis as a nanoproduct.

#### 2.2.2. Polymer Nanoparticles

Traditional polymers such as plastics, manmade fibers, rubber, and other polymers used in daily life have been unable to meet the special functions required in some fields, so advanced functional polymers with specific functions have been rapidly developed [81]. In the last century, researchers engaged in drug research and development began to pay attention to and design polymer therapy to improve related diseases [82]. With the rise of nano biological composites, polymer materials with different functional functions designed at nano-sizes are applied in delivery systems [83,84]. Self-assembled polymer systems such as polymer drugs [85], polymer protein conjugates [86], and polymer micelles [87] used for delivery at this stage can be connected with antibodies or fragments to achieve precise targeting of organs, tissues, and single cells. So, the application of polymer systems in the delivery of thrombolytic drugs shows great development prospects.

**Figure 6:**
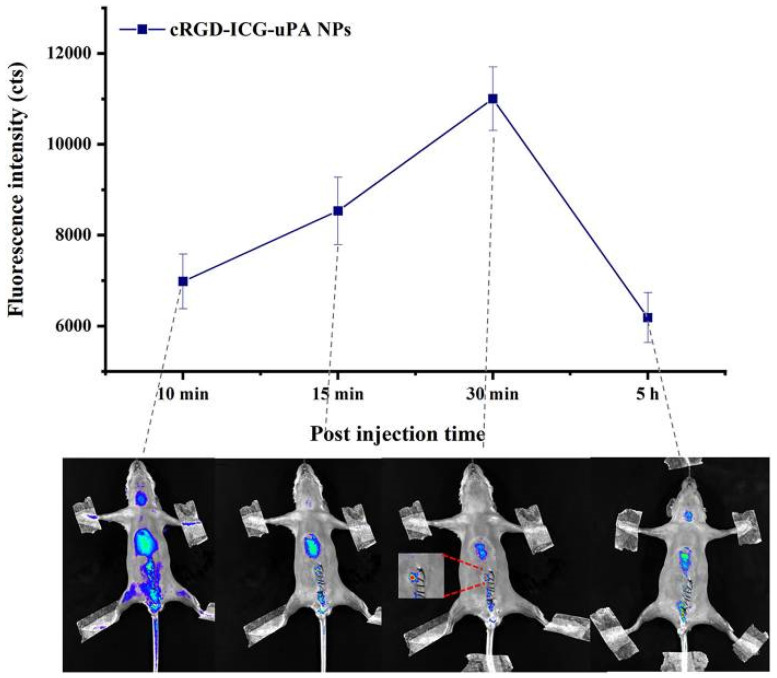
Average fluorescence intensity of thrombus after intravenous injection of cRGD-ICG-uPA NPs (20 U/g) on mouse mesenteric vascular thrombosis models, and in vivo fluorescence images at representative time points (*n* = 3). Reproduced with permission [96]. Copyright 2022, Frontiers.

Cationic polymers, such as poly (L-lysine) (PLL) [88], poly (acetylimine) (PEI) [89], and poly(2-(dimethylamino)ethyl methacrylate) (pDMAEMA) [90], are widely used as excellent nanocarriers for gene and protein drug delivery, and have been widely studied in gene and cancer drug delivery. Pan et al. hybridized pDMAEMA with other polymers as the core of drug delivery, and covalently linked RGD peptide and carrier to deliver lumbrokinase (LK) for targeted thrombolytic therapy. In the mouse model of carotid artery thrombosis, observation of the H&E staining section of the mouse carotid artery after treatment showed that LKM (LK-loaded micelles without RGD peptide) and LKTM (LK-loaded micelles with RGD peptide) could induce a reduction in the amount of thrombus in mice, but the residual thrombus in the LKM group was more than in the LKTM group. Due to the targeted administration, the bleeding time in the LKTM group was significantly shorter than in the LKM group. It is suggested that RGD peptide-conjugated polymer can effectively target thrombi, effectively reduce the risk of bleeding, and be used as a good thrombolytic drug delivery carrier [91]. The surface of dendrimers has a large number of exposed anionic, neutral, or cationic end-functional groups, which can produce hydrophilic or hydrophobic properties. Based on this feature, the problems of poor solubility and stability of drugs can be improved. The reaction sites provided by the end-functional groups can be used to bond drug molecules. When triggered by pH, enzymes, temperature, etc., at the lesion location, the covalent bond between the carrier and the drug is broken to achieve drug release [92,93]. Wu et al. synthesized a poly-L-lysine dendrimer (PLLD) to deliver nattokinase (NK) to reduce the effect of the in vivo environment on NK activity. Through the design of the carrier, NK/PLLD is positively charged, and the electrostatic interaction is used to penetrate into the negatively charged thrombus, so as to improve the thrombolytic efficiency. The tame substrate method showed that NK/PLLD nanocomposites still had high relative activity (more than 85%) at pH 6.0–9.0. In the in vitro thrombolysis experiment, the free NK solution reached 50% at 4 h, and the thrombolysis rate of NK/PLLD reached 50% at 12 h, which verified the slow-release effect of NK/PLLD nanocomposites on thrombolytic drugs, reduced the risk of massive bleeding caused by rapid release, and prolonged the circulation time of NK in vivo [94]. Among all biomaterials, polylactic glycolic acid (PLGA) has been recognized by many researchers as the most promising material in drug delivery and tissue engineering due to its excellent biodegradability. It can provide stealthy and effective biological interaction through surface modification of the material, and the design of its internal structure can have the feasibility of slow release [95]. Zhang et al. used an amphiphilic PEG-PLGA polymer as the core of the nanocarrier to create a new urokinase (uPA) indocyanine green (ICG, photosensitizer, used for phototherapy and fluorescence imaging) complex (ICG@uPA) encapsulated in cRGD peptide-modified polymer nanoparticles (cRGD-ICG-uPA NPs), which can achieve targeted thrombus photothermal synergistic therapy. In the in vitro release experiment, within the first hour, the cRGD-ICG-uPA NPs exhibited a relatively rapid release of ICG@uPA. However, most of the ICG@uPA exhibited a sustained release effect, lasting up to 24 h, with a final release rate of up to 80%. In the study of a mouse mesenteric vascular thrombosis model, it was found that cRGD-ICG-uPA NPs could rapidly accumulate at the thrombus site within 15 min after administration (Figure 6). After 24 h, no nanoparticles were detected in the whole body, as they may be gradually discharged from the body. The thrombus ablation rate of the cRGD-ICG-uPA NP group was 17% higher than that of the uPA group at one-third of the uPA dose. After 808 nm laser irradiation, the thrombus treated with nanoparticles decreased to 14% of its original size. The results show that this collaborative thrombolytic strategy can significantly improve the accuracy and efficiency of thrombolytic therapy, and its metabolic clearance characteristics bring more possibilities for clinical application [96].

Polypyrrole (Ppy) has excellent stability, conductivity, and good absorbance in the near-infrared (NIR) range. However, due to its hydrophobic nature, it has not been widely used in the biomedical field. In recent years, the hydrophobicity of Ppy has been improved by surface modification. With its excellent photothermal behavior and good biocompatibility, it has been studied for local hyperthermia of cancer [97]. The nanocarrier of ethylene glycol chitosan can express a specific response to the acidic microenvironment of pathological tissues [98]. Lu et al. designed and prepared thrombolytic nanoparticles (GCS-PPY-H NP) for dual-targeted photothermal therapy based on this advantage and combined with the high affinity of heparin for P-selectin (the basic and main target of blood clot formation). The fluorescence intensity of Cy5-labeled nanoparticles at the inflammatory site (pH~6.6) was observed to verify whether heparin mediates the thrombus targeting of nanoparticles. The results showed that the fluorescence intensity of heparin-modified group at pH 6.6 was significantly higher than that of the unmodified group. In vitro thrombolysis experiments indicate that GCS-PPY-H, when treated with NIR, exhibits effective thrombolytic therapy. In in vivo mouse experiments, the fluorescence intensity of the GCS-PPY-H group increased by 1.6 times compared to the non-heparin group, demonstrating superior thrombus homing capabilities [99]. The satisfactory thrombus targeting ability of this particle combined with photothermal thrombolysis has the potential to achieve nanomedical transformation. There are a large number of activated macrophages in atherosclerotic plaque, and cationic polymer can activate macrophages, so it is feasible to realize the targeted delivery of thrombolytic drugs by using the targeted movement of macrophages to the thrombus [100,101]. Inspired by this principle, Burnouf et al. prepared polypyrrole and cationic polymer polyethyleneimine (PEI) nanocomposites (PPy-PEI NCs) to deliver the thrombolytic agent lumbrokinase. The nanoparticles utilize the endocytosis of cations by monocytes/macrophages and the thrombus targeting mechanism of macrophages and the excellent photothermal properties of polypyrrole materials combined with thrombolytic agents to dissolve fibrin clots. In the experiment of detecting the ablation effect of NIR-treated nanocomposites on fibrin clots, as expected, the fluorescence intensity of the fibrin clots in the NIR-treated nanocomposite group gradually decreased. The specific accumulation of nanocomposites in the femoral vascular thrombus of rats was detected by preclinical in vivo optical imaging system (IVIS), which is speculated to be due to the specific targeting caused by the homing movement of macrophages after the uptake of nanocomposites. In the rat femoral vein thrombosis model, the thrombolytic effect of fluorescent labeled nanocomposites (Cy5-NCs) treated with NIR was tracked by histological imaging technology. The results showed that the thrombus size was significantly reduced, which may be due to the accumulation of macrophages at the thrombus site and the effect of NIR treatment on photothermal damage. Although the uptake and delivery mechanism of macrophages provides portability for the thrombus-targeted movement of nanocarriers, experimental studies have found that macrophages release a large amount of ROS in the high-temperature environment generated by NIR irradiation. As mentioned above, ROS have an important impact on the reformation of thrombi [102]. However, this proof-of-concept study provides a theoretical basis for our future study of local hyperthermia in the treatment of thrombosis and prevention of thrombosis. This product can graft L-arginine and other substances on the polymer that can react with ROS but do not produce harmful factors. By utilizing a certain stimulus to separate L-arginine from the polymer and allowing it to react with ROS, the concentration of ROS is reduced, minimizing the negative reactions generated by its thrombolytic mechanism.

#### 2.2.3. Hydrogel Nanoparticles for Thrombolytic Drug Delivery

**Figure 7:**
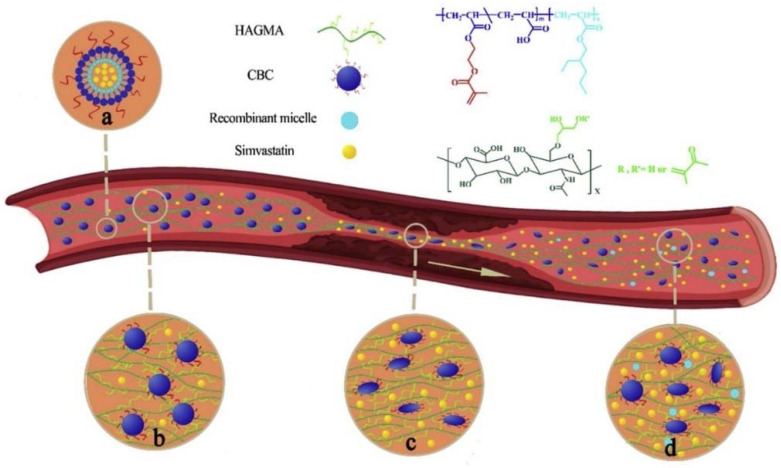
Schematic of a hydrogel drug delivery system capable of releasing drugs in response to mechanical stress at atherosclerotic plaques. The letters “a–d” in the figure caption denote different structures formed by the nanocarrier at various locations within the vascular system. Reproduced with permission [114]. Copyright 2019, Elsevier.

Hydrogel is a three-dimensional polymer network that can absorb a large amount of water and biological fluid. As early as the 1960s, gel that could be used for biological purposes appeared. Based on the hydrophilicity and high water content of hydrogels, a large number of researchers are committed to promoting the development of “intelligent” hydrogel technology. Nowadays, hydrogels are widely used in various branches of biomedicine, such as tissue engineering, drug delivery, cell delivery, biological dressings, and other fields. Micro/nano-level hydrogels have been used in the field of drug delivery to improve the lifecycle and precise controlled release of drugs because they take into account the characteristics of hydrogels and micro/nanoparticles [103,104,105,106]. In the case of ischemic stroke, even if the blood vessels are successfully reconstructed, the clinical symptoms of the patients do not change, which may be related to the failure to establish cerebral microcirculation. Ding et al. considered that the hypoxic conditions created by the ischemic process of ischemic stroke can lead to the low-pH microenvironment of infarcted brain tissue, so they crosslinked the protein and polymer chain through low-pH benzamide to prepare PEG-UK nanogel that can respond to the administration at pH~6.5 of infarcted brain tissue. Within the time window (about 4.5 h after stroke attack), the rats in the PEG-UK+UK group and the free UK group were, respectively, administered to the ischemic stroke model of rats. The results showed that the infarct volume of the PEG-UK+UK group was about twice that of the free UK group. In the treatment beyond the time window, the infarct volume fraction of the PEG-UK+UK group was 33.43 ± 6.51% within one hour of occlusion, which was similar to that of the free UK group. The relative infarct volume fraction of the PEG-UK+UK group was 13.55 ± 3.23% after 6 h of occlusion; the neurological scores were significantly improved. In the rat model of middle cerebral artery occlusion, the high-efficiency thrombolytic ability of PEG-UK preparation in response to a pH value of about 6.5 was confirmed. The results of both in vitro *and* in vivo research models indicate that utilizing the low-pH microenvironment created by ischemic stroke for pH-responsive drug release exhibits significant thrombolytic effects, effectively prolonging the therapeutic time window for thrombolysis in acute ischemic stroke, and potentially rescuing damaged neurons [107]. Zenych et al. also conducted relevant work on the treatment time window in ischemic cerebral infarction disease, using fucoidan (a naturally occurring algal-derived sulfated polysaccharide, a cheap and high-quality P-selectin targeting ligand, which has become part of the contrast agent for diagnosing atherosclerosis in the European Union [108,109]). The improved dextran (a non-toxic, biocompatible, biodegradable, and highly hydrophilic biopolymer [110]) hydrogel polysaccharide submicron combined with alteplase overcomes the problems of cerebral infarction and blood–brain barrier permeability after ischemia [111]. Due to the uncontrolled production of thrombin caused by infection, the production of thrombin makes fibrin deposit in the microvascular system, and the formation of microthrombosis consumes coagulation factors, which can start fibrinolysis and lead to diffuse hemorrhage, resulting in diffuse intravascular coagulation (DIC) [112]. Thrombosis and hemorrhage can occur at the same time; therefore, it is very important to explore a two-way treatment strategy that can dissolve thrombi and control bleeding. Mihalko et al. developed a core–shell nanogel (FSN) with fibrin specificity to balance the contradiction between dissolving microthrombosis and controlling bleeding. tPA-FSN and normal saline were, respectively, injected into the DIC rodent model to observe the microthrombosis in the heart, lung, and liver. It was found that the microthromboses in the heart, liver, and lung were significantly reduced after treatment with tPA-FSN. Platelet counts were performed on the animals treated with FSN and tPA-FSN. After treatment with tPA-FSN, the platelet count returned to the normal animal level, indicating that the consumption of coagulation factors in DIC was restored. When the nanogel was added to the plasma samples of patients with DIC, the addition of tPA-FSN made the clot structure more uniform, and the fiber density was reduced. A series of data shows that the nanogel has the potential to be used in the future to crack the abnormal clots in the microcirculation of patients with DIC and improve their coagulation dysfunction [113]. A variety of responsive nanogel delivery systems have been developed, including the “smart” nanogel stimulated by temperature, light, magnetic field, pH value, and so on. There are few reports on the in-depth study of the mechanical stress response of smart hydrogel at the thrombus. Yao et al. developed a composite hydrogel drug delivery system (HACBC) for atherosclerosis, which is mechanically stress-sensitive and can target thromboinflammatory macrophages (Figure 7). In the simulated vascular dynamic model, HACBC did not show mechanical stress-sensitive targeting in dynamic vessels with normal flow. When the drug-loaded HACBC was used to treat the vasculature with a luminal occlusion degree of 55%, and its drug release effect was tested, it was found that the drug release rate at 4 h reached 17.9%, which was 9.5% higher than the normal dynamic release rate. At 12 h, the drug release rate reached 31.2%, indicating that HACBC exhibits significant mechanical sensitivity. In the rabbit arterial embolization model experiment, the embolized rabbits were injected with HACBC and orally administered simvastatin solution (regulating blood lipid count and then treating atherosclerosis). The results showed that compared with the obvious thrombosis in the simvastatin group, there was only a small amount of adhesive thrombosis in the HACBC group. After measuring the length of the thrombus in the blood vessels and weighing, it was found that the thrombus length in the experimental group injected with HACBC was about 50% of that in the non-administration group, and the mass was reduced by as much as two times. The model of inflammatory macrophages injected with HACBC was detected by fluorescence imaging, and the significant red fluorescence in inflammatory macrophages proved its clear targeting effect on inflammatory macrophages. It is verified that the hydrogel is a dual-response intelligent hydrogel with mechanical stress sensitivity and cell targeting. The idea and preparation of this hydrogel provides a new treatment for atherosclerosis [114]. The essence of a thrombus is an insoluble platelet fibrin clot with a stable structure. Unknown factors activate platelets, which directly cause blood fibrin and platelets to aggregate and adhere to the intima of blood vessels to form a thrombus. Endothelium-derived NO can inhibit the adhesion of platelets to the sub-endothelium, prevent the activation and aggregation of platelets, and then prevent the formation of thrombosis. NO is easily cleared by oxyhemoglobin and myoglobin, which limits its effectiveness in reaching the target of vascular intima. Hosseinnejad et al. prepared highly hydrophilic nanogels (NOrel-NGs) crosslinked by a diselenide bond and used the diselenide bond to catalyze and maintain the endogenous NO production of physiological NO donor (S-nitrosoglutathione), thereby inhibiting platelet activation. This provides ideas for antithrombotic treatment and prevention of thrombosis [115]. The hydrogel, responsive to mechanical stress, releases thrombolytic drugs in response to the specific shear stress environment at the surface of thrombi. Moreover, this hydrogel system can target thrombus-associated inflammatory macrophages, thereby reducing inflammatory mediators at atherosclerotic plaques at their root, significantly minimizing the risk of arterial thrombus recurrence. In theory, it is suitable for embolic diseases with a small but not completely blocked lumen diameter.

### 2.3. Biologically Inspired Delivery of Biomimetic Nanoparticles

**Figure 8:**
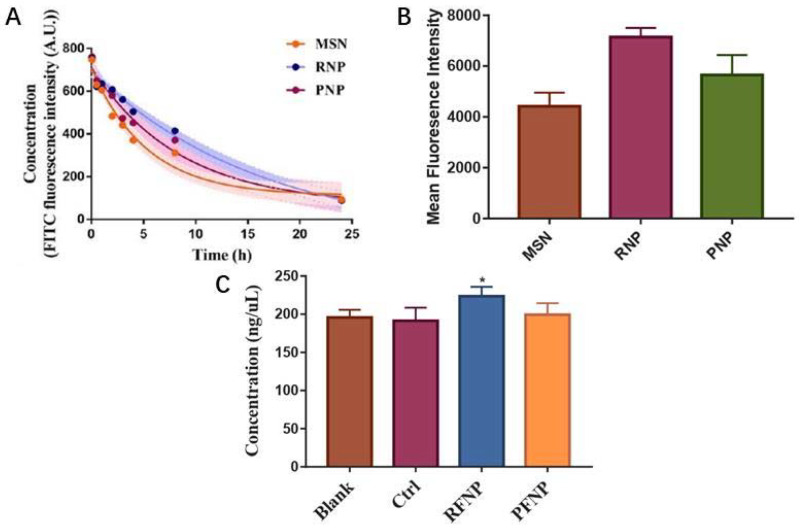
(**A**) Pharmacokinetics study of nanoparticles in blood circulation on rat; (**B**) the mean fluorescence intensity of the binding ability of nanoparticles to blood clot ex vivo; (**C**) D-dimer ELISA kit was used to detect the content of D-dimer in serum with PBS, RFNP, and PFNP injection, * *p* ≤ 0.05. Reproduced with permission [126]. Copyright 2020, ACS Publications.

The concept of imitating the structure and function of organisms or natural materials in nature to cope with the current technical difficulties has attracted extensive attention in the scientific community. The application of bionics principles in biomedicine, environmental engineering, and energy has achieved remarkable results. Among them, biomimetic membrane technology has developed into a relatively mature green technology [116,117,118,119]. The cell is the basic structural unit of life, so researchers were inspired to build a bionic nanodelivery system coated with cell membrane as a camouflage layer to escape the non-specific clearance of the immune system [120,121]. Based on the fact that cancer cell membrane-coated nanoparticles can be homogeneously combined with tumors of the same cell origin [122], researchers applied thrombolytic nanoparticles coated with platelet membrane and red blood cell membrane in the diagnosis and treatment of cardiovascular diseases to improve the ability of targeting thrombi and long-term circulation in vivo. Yu et al. designed a biomimetic nanovesicle that encapsulates melanin nanoparticles and tPA by platelet membrane (tPA/MNP2@PM,tPM), and used NIR-mediated photothermal treatment of melanin nanoparticles to lead to the rupture of nanovesicles, so as to achieve targeted controlled release and local photothermal thrombolysis at the same time. In clinics, about 40% of patients are disabled due to reperfusion after ischemic stroke, while melanin nanoparticles (4.5 nm) can clear free radicals and inhibit inflammatory reaction through the blood–brain barrier to avoid reperfusion injury after cerebral ischemia. In the mouse model of middle cerebral artery occlusion, Nissl staining was performed on the cerebral infarction site. It was found that tMP (NIR) treatment can increase the number of Nissl bodies (Nissl body reduction indicates nerve cell damage) and has a strong therapeutic effect on cerebral infarction. During reperfusion after thrombolysis, tMP (NIR) inhibits the expression of microglia and astrocytes, indirectly clears free radicals, and inhibits further damage from inflammation [123]. The research group of He et al. noticed that breast cancer cells (4T1 cancer cells) can easily achieve brain metastasis through the blood–brain barrier. Inspired by this, 4T1 cancer cell membrane was encapsulated with antioxidant and anti-inflammatory agent succinic butanediol (SCB), which was released in the way of pH response at the site of cerebral ischemia to reduce the inflammatory response after treatment of ischemic stroke. [124]. The neutrophil membrane-encapsulated nanoparticles can further precisely home in on the infarct core rather than normal brain tissue [125]. Compared with small molecules penetrating the blood–brain barrier, natural cell-derived membranes such as cancer cell membrane may have a better effect on encapsulating nanoparticles. The encapsulation of blood cell membrane retains the ability of immune escape and long circulation time. The erythrocyte membrane and platelet membrane also have the natural characteristics of interacting with fibrin and affecting thrombosis. Chen et al. explored and investigated the thrombolytic effect of two drug delivery regimens: red blood cell membrane-encapsulated fullerenol-loaded mesoporous silica nanoparticles (RFNPs) and platelet membrane-encapsulated nanoparticles (PFNPs). The blood circulation time of RFNPs and PFNPs was prolonged by 11.25 h and 5.85 h, respectively. The encapsulation of erythrocyte membrane had a more significant effect on the long circulation of nanoparticles (Figure 8A). Through the quantitative experiment of RNP- and PNP (both non-fullerenol-loaded nanoparticles)-anchored blood clots, it was found that the adhesion effect of RNPs was significantly increased by 61% compared with free mesoporous silica nanoparticles (MSNs), while PNPs only increased by 28% (Figure 8B). In the rat model of carotid artery thrombosis, RFNPs and PFNPs were used to treat left carotid artery thrombosis and test the level of D-dimer (a fibrin degradation product) in serum. The results showed that the level of D-dimer in the RFNP group decreased from 5.40 ± 0.67 mg to 2.76 ± 0.29 mg, while that in PFNP group was 3.86 ± 0.72 mg, and RFNPs significantly increased the level of D-dimer in serum compared with the PFNP group (Figure 8C); this index showed that the fibrinolytic effect of RFNPs is superior. A number of results show that the thrombolytic effect of nanoparticles encapsulated by erythrocyte membrane may be better than that of platelet membrane [126].

Although the nanodelivery system improves the blood circulation time of thrombolytic drugs, the drugs are only retained on the surface of the thrombus after release, and it is difficult to penetrate into the thrombus, which has a certain impact on the ideal therapeutic effect. Chen et al. developed a self-assembled combined phototherapeutic platelet nanomotor, cotton ball platelet (PLT), and poly (3,4-ethylenedioxythiophene) (with good near-infrared absorption and photothermal and photoelectric conversion characteristics, PEDOT) platform (P6@PEDOT @PLT) for phototherapy to deliver synthetic peptides derived from hirudin (P6) for noninvasive thrombolysis and anticoagulation therapy. The platform can generate localized high temperatures and electrical stimulation under NIR irradiation, polarizing M1-type inflammatory macrophages into M2-type anti-inflammatory macrophages and promoting the expression of vascular heat shock proteins (HSPs), while inhibiting the expression of plasminogen activator inhibitor-1 (PAI-1). This treatment not only dissolves thrombi but also repairs damaged blood vessels to prevent re-embolization of thrombi [127]. At present, the design of micro/nanomotors for thrombolysis is mostly driven by chemical reaction capacity and nanoparticles controlled by external electromagnetic field, followed by the homing movement of tangible components based on the principle of thrombosis, and there are few single cases of surface bacterial motors for antithrombotic formation. Xie et al. developed a tubular micromotor (^Fu^PDA_uPA_ @EcN) with *E. coli* (EcN) driving a polydopamine coating carrying uPA and fucoidan targeting thrombi for effective osmotic thrombolysis. In vivo experiments showed that the blood clot almost disappeared after 10 h of treatment with ^Fu^PDA_uPA_ @EcN. Driven by EcN, the blood perfusion rate reached 95.4%. ^Fu^PDA_uPA_ @EcN prolonged the half-life of uPA and the bioavailability was more than 10 times higher than that of free uPA. The tail hemorrhagic test found that there was no significant difference in the levels of red blood cells, white blood cells, platelets, or hemoglobin. The analysis results showed that there were no other complications (hemolytic anemia, acute infection, or bone marrow dysfunction) [128]. In 2017, recombinant adenovirus (AAV) drug delivery for cancer treatment was approved for clinical application, which proved the feasibility of its viral drug delivery and has become a potential treatment for cancer treatment. However, the cases of virus application in the field of thrombus diagnosis and treatment are relatively rare [129,130]. In order to effectively fight and treat thrombosis, it is particularly important to develop a method that can accurately locate the location of thrombus lesions and monitor the thrombolysis. Wen et al. developed and studied a biological nanoprobe based on plant virus to recognize thrombosis, chemically designed the shape and surface of plant virus (modified the binding peptide targeting fibrin), and evaluated the targeting effect of plant virus shape and surface ligand (fibrin-binding peptide GPRPP) using dual-mode optical and magnetic resonance imaging. Compared with the non-targeted control PEG-TMV, GPRPP-TMV particles showed a 9.5-fold binding force to thrombus. Icosahedral (spherical) cowpea mosaic virus (TMV) has more obvious thrombus attachment than slender rod tobacco mosaic virus (CPMV). In vivo studies have confirmed that the shape of viral nanoparticles contributes significantly more to thrombus targeting than targeted ligands [131]. By improving the shape of nanoparticles, the ability of thrombus-targeted drug delivery can be further improved. The application of viral vectors in thrombolytic drug delivery shows great potential in the future.

## 3. Biosafety Assessment of Nanomaterials

When nanomaterials (NMs) enter the field of cardiovascular diagnosis and treatment, they effectively change the traditional procedures for the diagnosis, monitoring, and treatment of some cardiovascular diseases. However, due to the small size and large surface area of nanoparticles, nanomaterials, and nanoagents, their interactions with biological systems in most cases cause adverse effects that are not easy to find in the short term compared with macro materials [132]. In order to safely apply nanomaterials, it is necessary to thoroughly evaluate the ideal and adverse reactions. When the existing definition of “toxicology” by the Toxicology Society is applied to nanomaterials, nanotoxicology describes the adverse effects of nanomaterials on organisms and the prevention and improvement of such adverse effects. Nanotoxicology mainly studies the physiology, pathology, and biomolecular mechanism of nanomaterials. A review of the comprehensive toxicological analysis of NMs has been published in some journals. The paper makes a necessary safety analysis on the application of NMs in the diagnosis and treatment of thromboembolic diseases [133].

For the biological safety of nanoparticles, researchers often focus on the cytotoxicity, blood compatibility, immunotoxicity, biodistribution, and degradation in vivo and in vitro of nanoparticles. The dosage of nanoparticles; the size, shape, and composition of nanoparticles; and the route of exposure or administration may affect organisms. Nanoparticles used for thrombolysis itself can also cause platelet aggregation, and then induce thrombosis. Radomski et al. reported that carbon nanomaterials can easily induce platelet activation and aggregation in vivo. Interestingly, however, platelet aggregation is induced by nanotubes rather than nanospheres [134,135]. This phenomenon indicates that the shape of nanoparticles indirectly affects the formation of thrombosis. Knudsen et al. found that the toxic reactions of positively and negatively charged nanoparticles to the rat brain were different, and intravenous administration of high doses of anionic and cationic NPs for 7 consecutive days did not show systemic toxicity. Direct administration of anionic NPs into the lateral ventricle did not cause any changes in the administration site. However, cationic administration at the same site showed local infiltration of phagocytes and inflammatory cells, neuronal loss, astrocyte proliferation, and apoptosis, resulting in histological changes. The study suggests that cationic particles may be toxic to brain tissue [136]. Special attention should be paid to the tissue toxicity to the brain when cationic particles are used to treat ischemic stroke and reperfusion loss through the BBB. In addition, studies on functionalized modification of positively charged chitosan on the surface of AuNPs have found that functionalized AuNPs drive inflammation and decrease cell viability. The coupling of two seemingly non-toxic NP materials may induce strong cytotoxicity. Therefore, after the modification and deformation of AuNP, it is necessary to fully evaluate its overall performance [137]. MSNs with surface-rich silanols can bind to phosphatidylcholine in RBC membranes, which can easily cause bending and deformation of red blood cells, but this deformation feature is also influenced by nanoparticle size. MSNs contain a large number of siloxane bonds, which cause them to degrade into silicic acid in aqueous media and be eliminated in the body through the kidneys [138]. Silicon dioxide nanoparticles can affect the function of lysosomes, leading to autophagy, oxidative stress, and apoptosis in endothelial cells [139]. MNPs larger than 100 nm are retained in the liver and spleen through macrophage phagocytosis, which can effectively avoid clearance by the reticuloendothelial system by reducing the size of MNPs. The toxicity is mainly caused by the increase in ROS in host cells. Biological and chemical coatings are applied to the surface of MNPs to improve their distribution and stability in human blood flow, and to limit such nanotoxic effects [140]. Carbon nanomaterials (CBNs) are widely used due to their excellent photothermal properties, but they have a specific impact on the integrity and permeability of the endothelial barrier. CBNs can increase the permeability of endothelial cells. When vascular tension is impaired, excessive accumulation of interstitial fluid leads to vascular leakage and organ damage, further affecting the reformation of blood clots [141].

## 4. Discussion and Conclusions

At this stage, some progress has been made in the application of nano-functional materials in the field of thrombus diagnosis and treatment (Table 1). 

Using the biochemical characteristics of the thrombus itself, the surfaces of nanoparticles are modified with molecules that can specifically target the location of the thrombus, and active targeted thrombolysis is carried out with antigen antibody binding as the main connection. The passive targeting of magnetic nanoparticles to the location of thrombus lesions in an alternating magnetic field is controlled, and dual-targeted thrombolysis has attracted the attention of scholars. The development of dual-targeted thrombolytic delivery system is no longer limited to the combination of magnetic and targeted peptides. The non-contact photoacoustic stimulation signal with high temporal and spatial resolution has become a new combination of dual-targeted delivery. In the treatment of thromboembolic diseases in the acute phase, researchers have sought two more effective solutions. One is to modify or modify the structure of nanocarriers to make nanoparticles dynamic. Second, the blood clot is transformed into a multi-channel structure by external stimulation to improve the retention time of nanoparticles in the lesion. The thrombolytic efficiency is greatly improved. At the same time, researchers also put forward corresponding solutions for ischemic tissue injury and thrombosis after recanalization, which can improve the prognosis of patients, in theory. However, there are still some problems that have not been solved. For example, under the high-speed shear force of blood flow in the body, the nanocarrier cannot achieve high-efficiency targeted enrichment at the thrombus lesion, and the residence time at the thrombus is short, which makes the drug release incomplete, and the effect is not concentrated, thus affecting the thrombolytic effect; the accuracy of passive thrombus targeting in deep veins needs to be further improved. However, the appearance of nanomotors improves retention time in the thrombus, and improves the penetration depth of nanoparticles, so that thrombolytic drugs no longer only act on the thrombus surface, which brings risks while improving thrombolytic efficiency. Thrombolysis from the inside to the outside may increase the risk of breaking large clots into small clots and then blocking the microvessels. The influence of substances from which the motor generates power on the physiological process of the body is not clear. In the part with large thrombus, a small thrombus is formed due to incomplete thrombolysis, and the microvessels are blocked when flowing through the microcapillaries, increasing the risk of bleeding. Although the existing nanocarriers have solved many problems, in order to make the nanocarriers have the characteristics of the integration of diagnosis, treatment, and other multifunctionality, a variety of exogenous molecules have been modified on the surfaces of nanocarriers. The modification of mesoporous silica carriers with too many exogenous molecules has greatly reduced the drug release effect. The biocompatibility, organ cumulative toxicity, and induced immune response of modified nanocarriers need to be further studied; the side effects induced by the leakage of thrombolytic drugs on nanocarriers should also be focused on; although the newly developed biomimetic nanocarriers have overcome the defects of some conventional nanodelivery systems, the possibility of scale-up production due to preparation problems is still unclear. At present, although a variety of multifunctional nanoparticles have been developed, none of them have been approved by the FDA for clinical application. The main reason is that the realization of multifunctional nanoparticles makes the preparation process of nanocarriers complex, and it is difficult to commercialize into clinical transformation. Moreover, multifunctional nanoparticles depend on external stimulation. However, the medical equipment that can realize such stimulation is expensive, grass-roots hospitals cannot achieve popularization, and it is still in the laboratory stage or clinical trial stage. In order to realize the large-scale production of nanocarriers, the use of intermolecular forces to spontaneously assemble or aggregate to form an orderly spatial structure can greatly improve the simplicity of the preparation process of nanoparticles, which may greatly promote the clinical transformation of nanodrugs. Although nanoparticles in the laboratory stage have almost no toxicity to the liver, kidney, and other organs, it is not fully known whether they will affect other physiological functions of the body in the human environment. Therefore, in the selection of nano-functional materials, biodegradable materials approved by the FDA should be selected to reduce unknown risks. We speculate that liposomes and magnetic nanoparticles may become the first batch of nanoparticles in clinical application in the future. In the future, the mainstream direction of nanomedicine in the treatment of diseases may no longer be the use of a single material as a carrier to deliver drugs for thrombolysis but rather the combination of natural drugs with special chemical properties as a carrier and thrombolytic drugs for collaborative treatment, no longer relying on external stimulation, but using the responsiveness of the thrombus microenvironment for autonomous thrombolysis. With the continuous progress of technology and the continuous expansion of application scenarios, perhaps nanorobots will play an important role in the field of cardiovascular therapy, and further enable nanotechnology to promote the development of precision medicine.

## Figures and Tables

**Table 1:** Different nanomaterials for thrombolytic therapy.

Nanocarriers	Advantages	Disadvantages	Therapeutic Effects
Mesoporous silica	🗸 High drug loading rate 🗸 Easy surface modification 🗸 Good stability	◆ Prone to drug leakage	● Increased thrombolysis rate ● Removed excess ROS
Magnetic nanoparticles	🗸 Directional motion under applied magnetic field 🗸 Good biocompatibility 🗸 Multifunctional diagnosis and treatment integration 🗸 Photothermal characteristics	◆ Prone to particle agglomeration ◆ May induce other immune responses	● MRI-assisted diagnosis ● Photothermal thrombolysis ● Excellent thrombolytic effect
Liposomes	🗸 Low immunogenicity 🗸 Clinically approved	◆ Low stability ◆ Low drug entrapment efficiency	● Ultrasound-assisted thrombolysis ● Reduced risk of bleeding
Polymer	🗸 Functional surface modification 🗸 Mostly biodegradable	◆ Stability needs to be further improved	● Outstanding thrombolytic effect ● Better metabolic clearance ● Excellent hyperthermia
Hydrogel	🗸 Responsive release drug	◆ Application in specific environment	● Improved neurological scores ● Increased drug release rate ● For improving coagulation dysfunction
Biomimetic nanomaterials	🗸 Long circulation in the body 🗸 Natural thrombus homing characteristics	◆ Low reproducibility ◆ Source of preparation ◆ Enlarge production possibilities	● Excellent thrombus monitoring ability ● Precise homing of infarct core ● Reduced inflammatory response

## Data Availability

No new data were created or analyzed in this study. Data sharing is not applicable to this article.
